# A Cellulosic Fruit Derived from *Nerium oleander* Biomaterial: Chemical Characterization and Its Valuable Use in the Biosorption of Methylene Blue in a Batch Mode

**DOI:** 10.3390/polym12112539

**Published:** 2020-10-30

**Authors:** Youssef O. Al-Ghamdi, Mahjoub Jabli, Raoudha Soury, Shahid Ali Khan

**Affiliations:** 1 Department of Chemistry, College of Science Al-Zulfi, Majmaah University, Al-Majmaah 11952, Saudi Arabia; yo.alghamdi@mu.edu.sa; 2 Chemistry Department, Faculty of Science of Hail, University of Hail, Hail 81451, Saudi Arabia; ra.soury@uoh.edu.sa; 3 Department of Chemistry, University of Swabi, Swabi Anbar, Khyber Pakhtunkhwa 23561, Pakistan; skhan@uoswabi.edu.pk

**Keywords:** *Nerium oleander* fruit, cellulose **I**, methylene blue, adsorption

## Abstract

Cellulose substrate waste has demonstrated great potential as a biosorbent of pollutants from contaminated water. In this study, *Nerium* *oleander* fruit, an agricultural waste biomaterial, was used for the biosorption of methylene blue from synthetic solution. Fourier-transform infrared (FTIR) spectroscopy indicated the presence of the main absorption peak characteristics of cellulose, hemicellulose, and lignin compositions. X-ray diffraction (XRD) pattern exhibited peaks at 2θ = 14.9° and 2θ = 22°, which are characteristics of cellulose **I**. Scanning electron microscopy (SEM) showed a rough and heterogeneous surface intercepted by some cavities. Thermogravimetric analysis (TGA) showed more than a thermal decomposition point, suggesting that *Nerium* fruit is composed of cellulose and noncellulosic matters. The pH_pzc_ value of *Nerium* surface was experimentally determined to be 6.2. *Nerium* dosage, pH, contact time, dye concentration, and temperature significantly affected the adsorption capacity. The adsorption capacity reached 259 mg/g at 19 °C. The mean free energy ranged from 74.53 to 84.52 KJ mol^−1^, suggesting a chemisorption process. Thermodynamic parameters define a chemical, exothermic, and nonspontaneous mechanism. The above data suggest that *Nerium* fruit can be used as an excellent biomaterial for practical purification of water without the need to impart chemical functionalization on its surface.

## 1. Introduction

Contaminated water can be successfully treated by either physical or chemical processes. This can be accomplished using inexpensive and biodegradable alternative adsorbents that decrease the treatment cost of contaminant elimination. In this framework, agricultural waste biomaterials received much attention due to their high availability in nature and low cost. Many biosorbents have been used to remove dyes from water, such as cotton stalks [1], potato wastes [2], peach shells [3], almond shells [4], mandarin peels [5], wood apple shells [6], grape fruit peels [7], peanut hulls [8], peats [9], rice husks [10], wood sawdust [11], peanut husks [12], pine sawdust [13], orange peels [14], banana peels, and coconut mesocarp [15].

Indeed, there are huge amounts of agricultural wastes that are produced on a daily basis. *Nerium oleander* plant, having a wide geographical distribution, has attracted the attention of researchers due to its use in various applications [16,17,18]. The fruits of *Nerium* are narrow capsules with a length of 5–23 cm. After maturing, they release soft seed fibers. The use of *Nerium* fruits and seed fibers as biosorbents of pollutants have not been reported in the literature. In this sense, only the work of Rajab et al. [19] has carried out such study; however, it was restricted to the possibility of utilizing oleander tissues as sorbent material. Our recent studies on *Nerium oleander* focused on the use of seeds modified with ethylenediamine and hydrazine for the sorption of acid dyes [20]. In addition, we studied the chemical characteristics, dyeing behavior, and abilities of these seed fibers to adsorb cationic dyes [21,22]. Recently, we used *Nerium oleander* leaves for the ecological synthesis of CuO nanoparticles, and the prepared particles were applied for the adsorption of methylene blue [23]. Here, the main purpose of the current work was, first, to characterize *Nerium oleander* fruit and, second, to further evaluate its adsorption capacity for methylene blue under several experimental conditions. Its adsorption performance was checked in relation to that reported in previously published agricultural biosorbents. FTIR, SEM, XRD, and TGA analyses were used to elucidate the functional group characteristics, crystallinity, morphology, and thermal behavior of *Nerium* fruit. Kinetic theoretical equations were used to understand the affinity between methylene blue as adsorbate and *Nerium* fruit as adsorbent. The biosorption mechanism on the biomaterial surface was evaluated using the common isotherms of Langmuir, Freundlich, Temkin, and Dubinin.

## 2. Experimental

### 2.1. Materials and Reagents

All chemical reagents used in the current work were of pure grade and used without further purification. Methylene blue, with a molecular weight of 319.85 g mol^−1^, was supplied by a Sigma-Aldrich market. Deionized water was used in diluted colored solutions. 

### 2.2. Preparation of Nerium oleander Fruit

**Figure 1:**
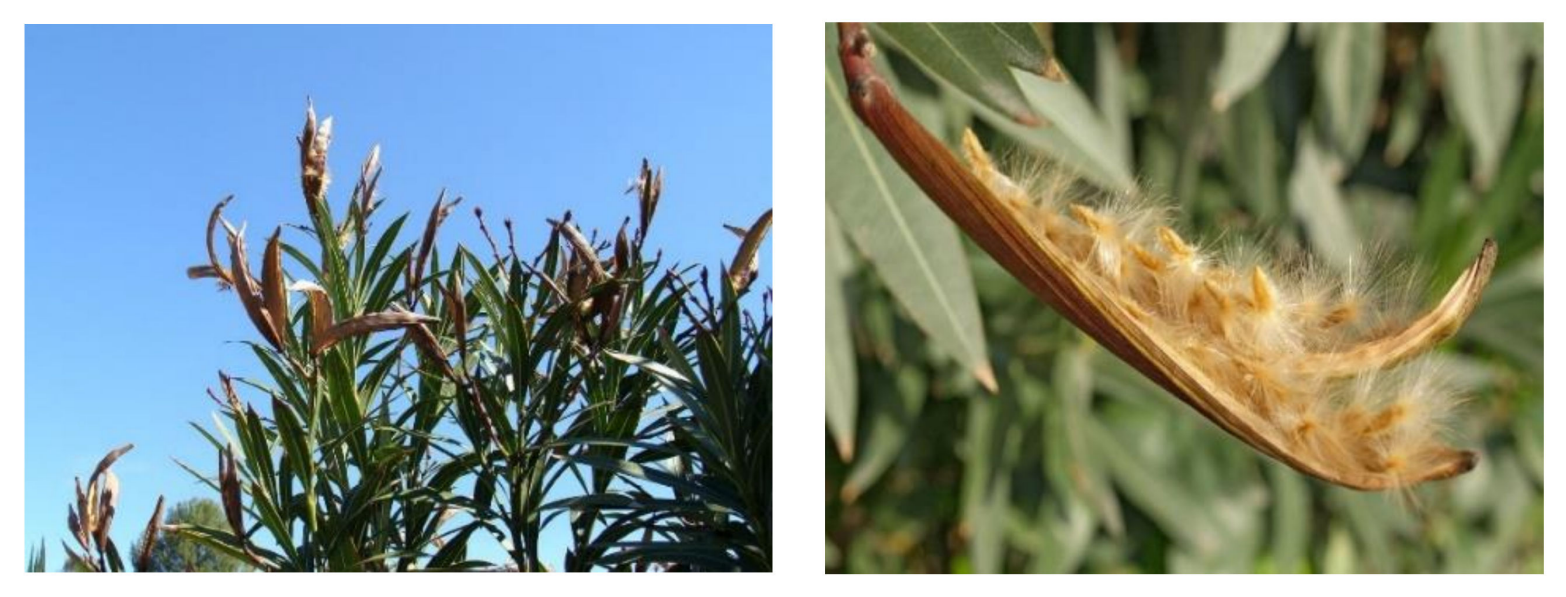
Photographs showing collection of *Nerium oleander* fruit as agricultural waste.

Ripe *Nerium oleander* fruit was cultivated during the period of May–June (Figure 1). Before being characterized and used as adsorbent, the biomass was thoroughly washed with water in order to remove adhering impurities, like sand particles and debris. The dried fruits were further washed with distilled water and dried in an oven at a temperature of 70 °C for a period of 24 h. The fruits were ground to small powders, washed with distilled water, and finally dried under the abovementioned conditions. Before adsorption investigation, the powdered fruits were stored in airtight bottles.

### 2.3. Characterization Instruments

To elucidate the chemical group characteristics of *Nerium oleander* fruit, an FTIR spectrum was obtained using a PerkinElmer model (Monastir, Tunisia). A SEM Hitachi S-2360N apparatus (Monastir, Tunisia) was used to observe the morphology of the studied powdered fruit, which was coated with Au by a vacuum sputter coater with a 20 kV accelerating voltage. X-ray powder diffraction was performed at ambient temperature using a PANalytical X’Pert PRO MPD apparatus (Malvern, UK). Thermogravimetric analysis (TGA) was performed in air flow at a heating level of 10°/min using a NETZSCH STA 449F3 equipment (Selb, Germany).

The point of zero charge (pH_PZC_) of powdered *Nerium* fruit was obtained by the addition of KNO_3_ solution: 0.1 g of solid biosorbent was immersed in 10^−1^ M KNO_3_ solutions (50 mL) at different pH values. The initial pH was maintained in the range 2–11 by the addition of either HCl or NaOH under magnetic agitation for 48 h. The final pH value was plotted versus the initial pH value. The pH_PZC_ was registered when the point pH_final_ = pH_initial_ [24].

### 2.4. Biosorption Experiments

Batch biosorption experiments were performed in Erlenmeyer flasks using 20 mL of methylene blue solution and 0.025 g of powdered *Nerium* fruit. The biosorbent dosages were studied in the range 1.25–7.5 g/L. The isotherm experiments were conducted at 19, 40, and 55 °C. The kinetic essays were studied in the range 0–120 min. After each experiment, the solutions were filtered through filter papers, and the absorbance of the remaining solution was determined spectrophotometrically at λ_max_ = 665 nm corresponding to the maximum absorbance of methylene blue. The adsorption capacity was calculated using the following equation:

(1) $$q\left(mg/g\right)=\frac{\left(c0-\mathrm{ce}\right)}{m}\times V$$

where c0 is the initial dye concentration, ce is the remaining solution, V is the used volume of dye for each experiment, and m is the mass of the used biosorbent.

To describe the biosorption mechanism of methylene blue using powdered *Nerium* fruit, the experimental results were correlated with the theoretical isotherms of Langmuir, Freundlich, Temkin, and Dubinin, which are generally used to clarify the mechanism of biosorption as well as the heterogeneity of the biosorbent surface [24].

## 3. Results and Discussion

### 3.1. Characterization of Nerium oleander Fruit

**Figure 2:**
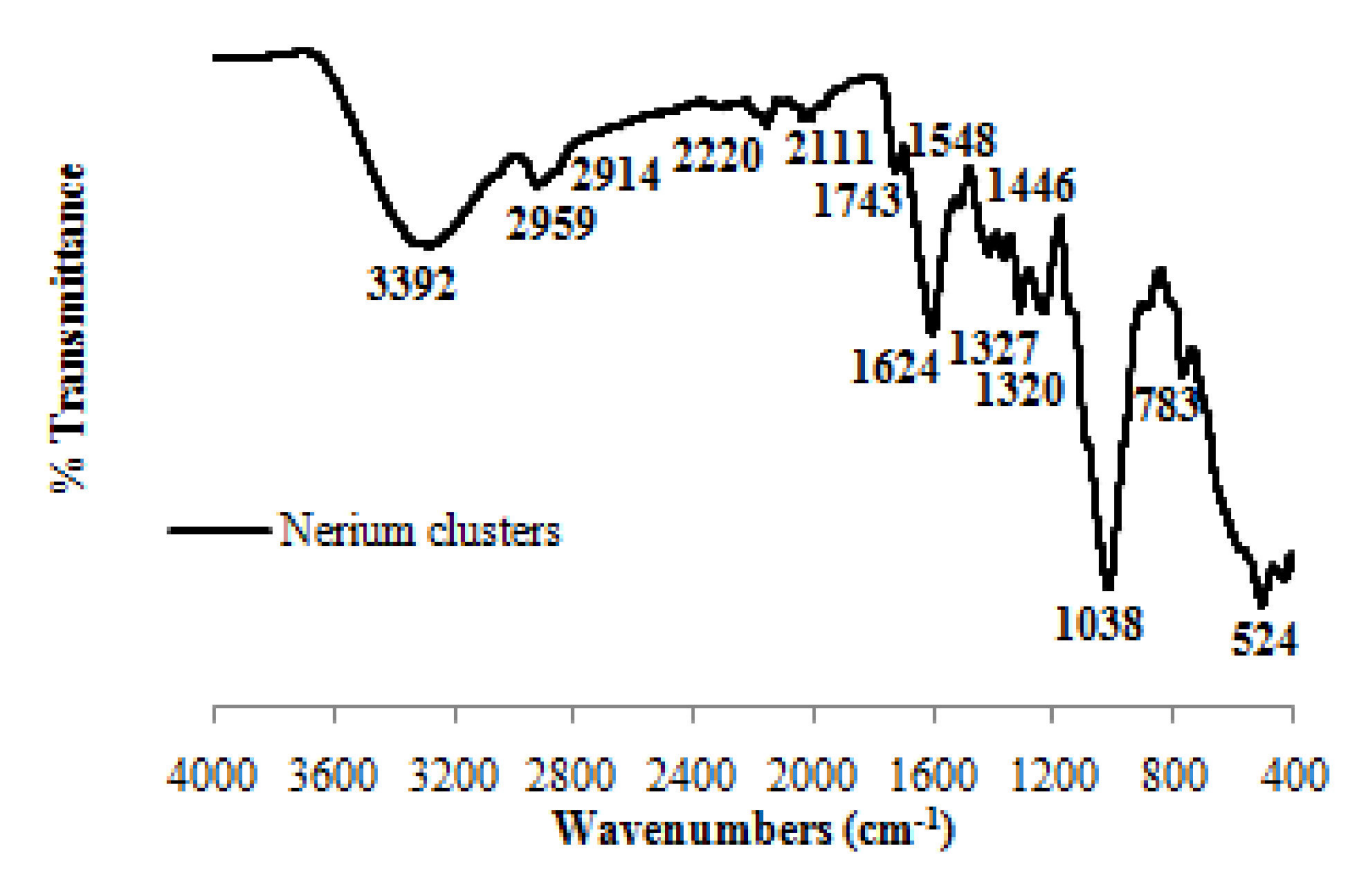
FTIR spectrum of *Nerium oleander* fruit.

**Scheme 1:**
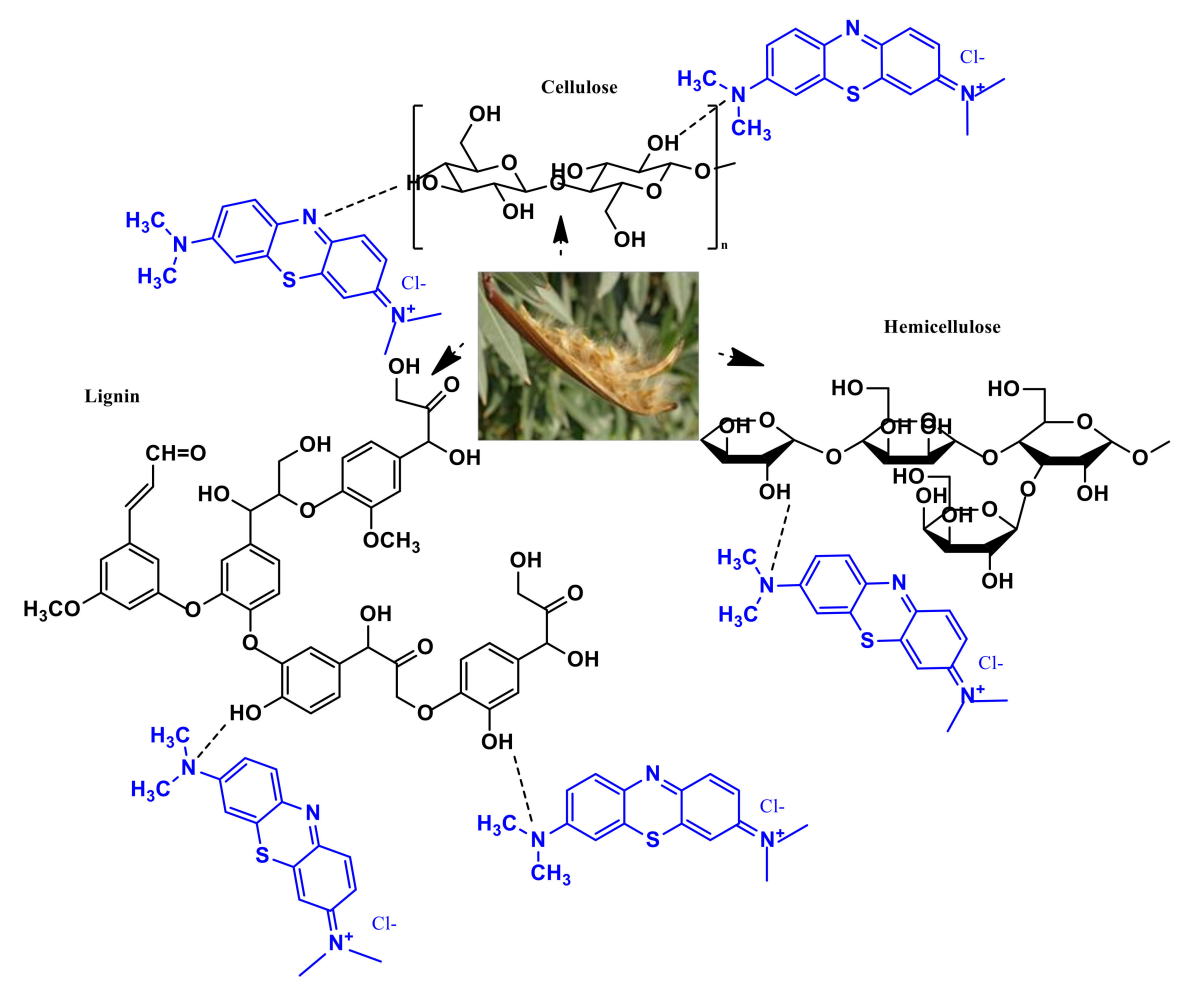
Proposed mechanistic pathway for interactions between methylene blue and *Nerium oleander* fruit surface.

FTIR spectroscopy can afford information about the reactive groups existing on the surface of *Nerium* fruit that provide adsorptive sites for methylene blue molecules. Figure 2 displays the main absorption peaks registered within the studied biomaterial. The broad intense absorption peak around 3392 cm^−1^ is attributed to the hydroxyl groups. The bands at 2959 and 2914 cm^−1^ correspond to C–H stretching of the –CH_3_ and –CH_2_ groups, respectively [25,26]. The peaks at 2100–2200 cm^−1^ show the presence of CO_2_ in normal air. The peak at 1743 cm^−1^ confirms the functional C=O group present in hemicelluloses [25,26]. The absorption peaks registered at 1624–1548 cm^−1^ are attributed to the C=C aromatic groups in the lignin [27,28]. The absorption peak at 1446 cm^−1^ is assigned to the angular deformation of the –CH groups in the cellulose structure [27]. The peak observed at 1320–1327 cm^−1^ corresponds to the angular deformation of the –CH groups in hemicelluloses [28]. The peak at 1038 cm^−1^ may be assigned to the C–O symmetric or asymmetric vibration (–C–O–C– ring) of the cellulose. The peak at 783 cm^−1^ is related to the out-of-plane angular deformation of the –CH groups in substituted aromatic rings [29]. The majority of these peaks indicate that *Nerium oleander* fruit is rich in massive oxygenous groups on its surface [26], which could be responsible for the interaction between methylene blue and the biomaterial through hydrogen bonding. In Scheme 1, a suggested mechanistic pathway for interactions between methylene blue and *Nerium oleander* fruit surface is depicted. Indeed, the hydroxyl groups of the biomaterial could easily react to the nitrogen atom of methylene blue through hydrogen bonding.

**Figure 3:**
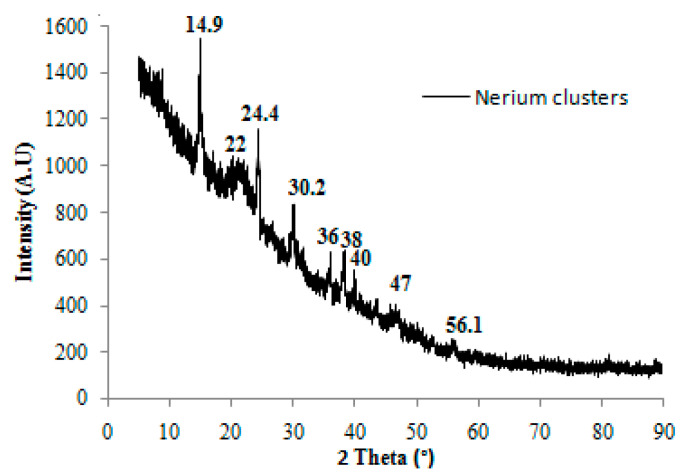
XRD patterns of *Nerium oleander* fruit.

Powder X-ray diffraction was used to describe the powdered *Nerium oleander* fruit by scanning in the range 10°–90° (2θ°) (Figure 3). The biomaterial exhibited the characteristic diffraction peaks at 2θ = 14.9° (amorphous phase) and 2θ = 22° (crystalline phase). According to the literature, these peaks are characteristics of crystal planes (110) and (200) of cellulose **I** [30,31]. The strong peak observed at 2θ = 14.9° indicates the presence of a high proportion of noncellulose compositions [32,33]. These main peaks were also observed in our previous paper [22] when reporting XRD patterns of the seed fibers encapsulated in the narrow capsules of *Nerium oleander* plant. The presence of other small peaks could be attributed to the existence of inorganic matters.

**Figure 4:**
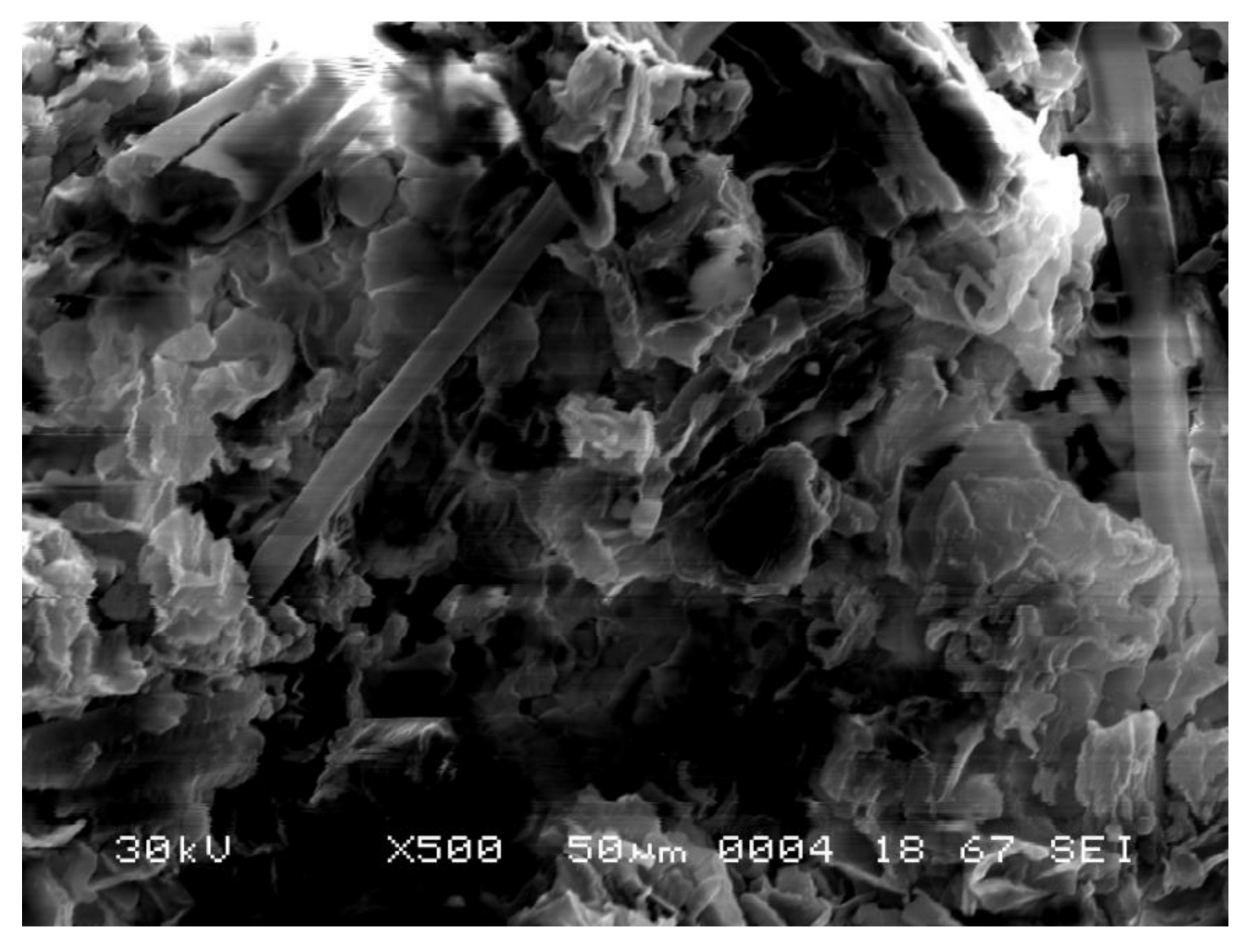

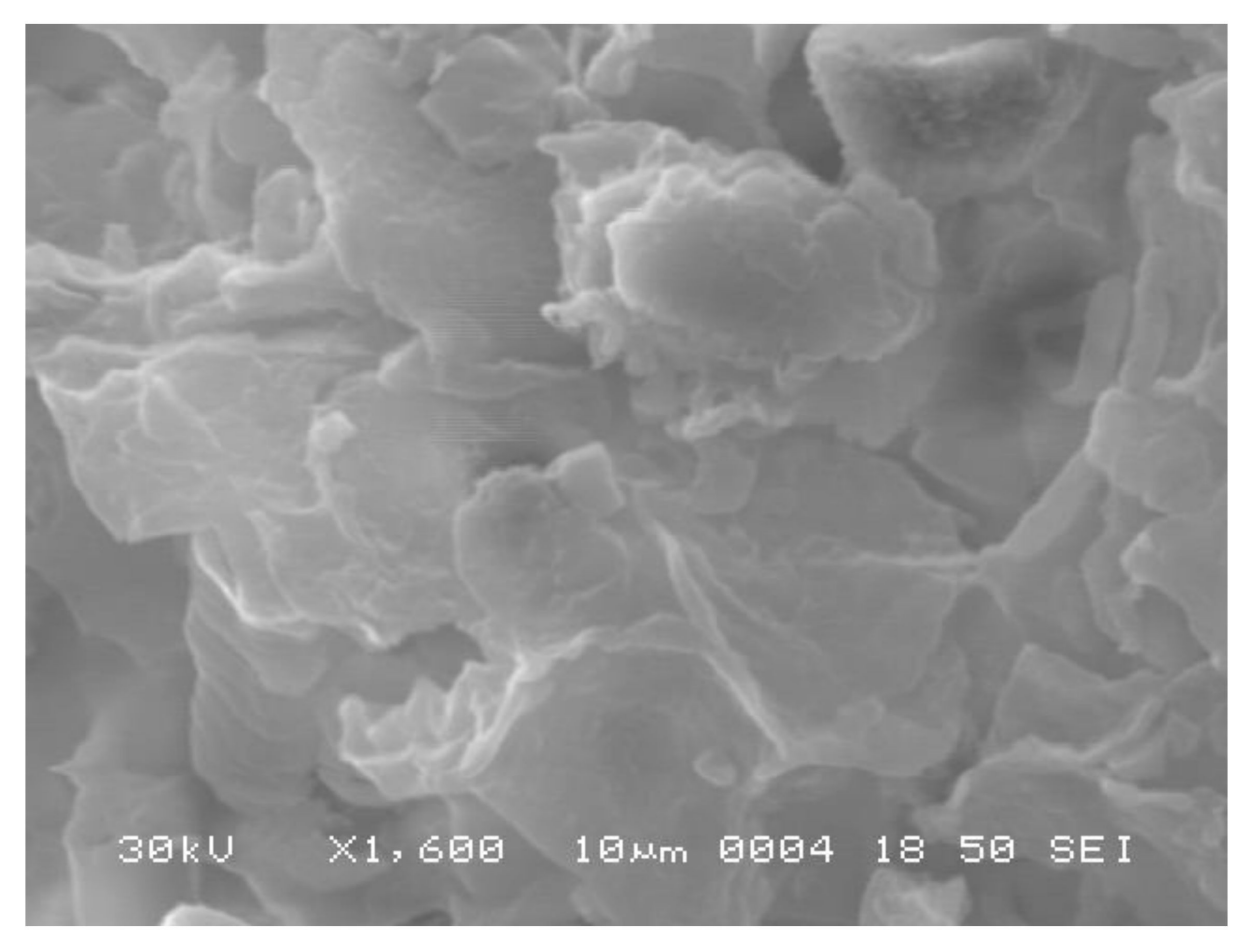
SEM images of *Nerium oleander* fruit.

SEM images (Figure 4), given at different higher magnifications (×500 and ×1600), showed a rough and heterogeneous surface of *Nerium oleander* fruit with the existence of some cavities. These precise morphological characteristics could easily facilitate the interaction between the studied solid biomaterial as biosorbent and methylene blue as adsorbate.

**Figure 5:**
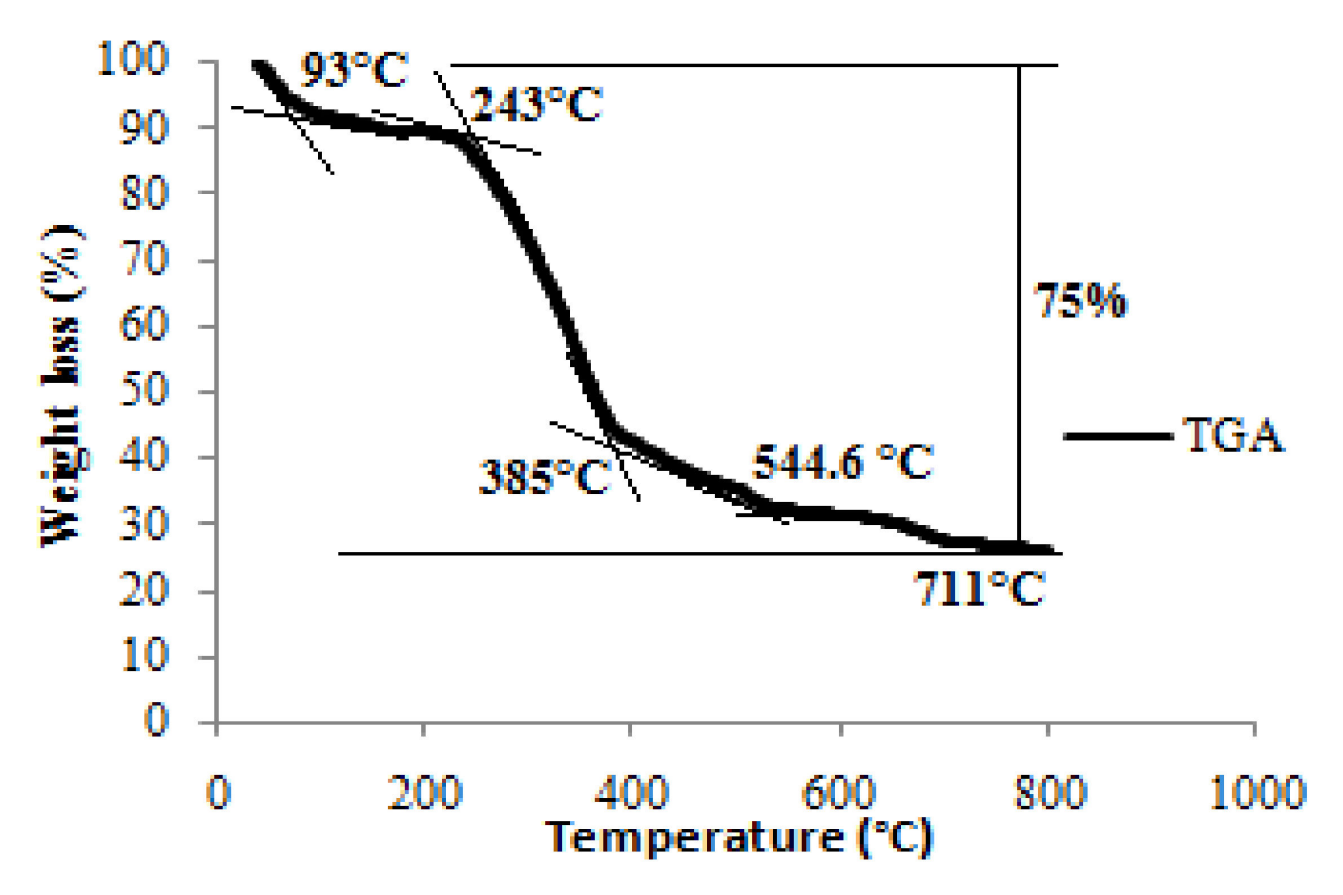
TGA curve of *Nerium oleander* fruit.

Thermal analysis was carried out to study the decomposition pattern and thermal stability of *Nerium* fruit. The TGA curve of *Nerium* fruit is depicted in Figure 5. Many thermal decomposition points were observed at different temperature values. The maximum mass loss reached 75%. This indicates that the studied biomaterial was composed of cellulose and noncellulosic matters. This behavior agrees well with the results gleaned from FTIR, XRD, and SEM analyses. A weight loss of 8% was observed at 93 °C, which could generally be ascribed to the moisture evaporation on the surface and inside the biomaterial [34,35]. The thermal decomposition observed at 243 °C could be assigned to the decomposition of the organic compounds, including cellulose and noncellulosic matters, found in the biomaterial [36,37]. The thermal decomposition at 385 °C could be due to the depolymerization and decomposition of glycosyl units [38]. The slight decomposition observed at 544–711 °C may be associated with the degradation of inorganic compounds existing in native fruits. 

### 3.2. Factors Affecting the Adsorption of Methylene Blue

#### 3.2.1. Effect of Experimental Parameters on Adsorption Performance

**Figure 6:**
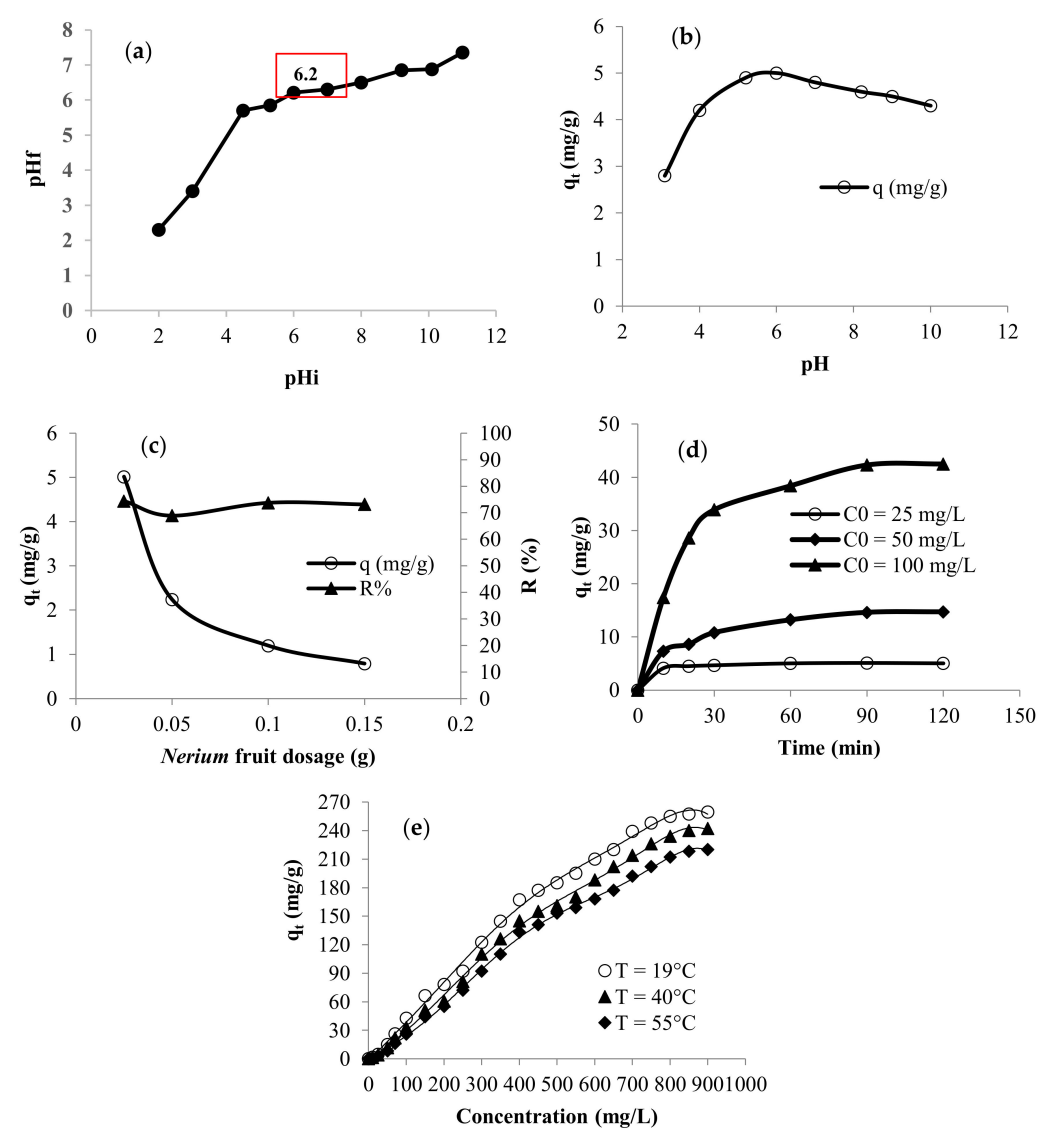
Influence of some experimental parameters on the biosorption of methylene blue using *Nerium* fruit: (**a**) plots of pH final versus pH initial, (**b**) effect of pH value, (**c**) evolution of adsorbent dosage, (**d**) variation of time, (**e**) change of methylene blue concentration, and temperature.

Figure 6a gives a plot of the initial pH against the final pH. The pH_pzc_ value of *Nerium oleander* fruit was determined to be 6.2. This finding suggests that the surface of the biomaterial was positive at pH < 6.2 [39]. However, it became negatively charged at pH > 6.2, therefore permitting an electrostatic attraction with the cationic methylene blue. Indeed, methylene blue dye can easily dissociate in water, giving MB^+^ and Cl^−^ ions [40].

The biosorption of methylene blue using *Nerium oleander* fruit was investigated in the pH range 3–11 (Figure 6b). The results indicate that a low biosorption capacity was reached at high acidic conditions. The highest adsorption capacity was achieved at pH 6, above which a decrease was observed. This finding agrees well with the pH_pzc_ determined experimentally (Figure 6a). Based on FTIR and XRD results, *Nerium* fruit has many oxygenous groups as adsorption sites on the surface, therefore allowing different trends at different pH values. Positive charges were acquired by the *Nerium* surface at an acidic pH due to protonation, and a negative charge was acquired due to deprotonation of oxygenous groups at alkaline conditions. At acidic pH values, the positively charged methylene blue ions opposed the positively charged *Nerium* surface, leading to low biosorption amounts. At higher pH values, the biosorbent surface became negative, favoring an electrostatic interaction with methylene blue. In this case, it was observed that the biosorption tendency of *Nerium* fruit increased with an increase in pH due to an increase in the deprotonation of the oxygenous groups of the adsorbent. Quite similar behaviors were observed and reported in other previous published works [24,41,42,43].

Figure 6c indicates that the adsorption of methylene blue using *Nerium* fruit was affected by the change of adsorbent dosages ranging from 1 to 6 g L^−1^. The maximum adsorbed amount of methylene blue was observed with 1 g L^−1^ of *Nerium* fruit, and further added biosorbent decreased the adsorption of methylene blue. The high degree of removal of methylene blue registered at the lowest adsorbent dosage may be attributed to the large adsorption sites of the *Nerium* fruit surface [44]. 

The plots representing the evolution of adsorbed methylene blue versus contact time (Figure 6d) indicate that about 80% of dye molecules were removed within the first 30 min and attained equilibrium at 90 min. The explanation of this behavior is that, at the initial period of time, many vacant active sites were available at the surface of *Nerium* fruit, which were responsible for the rapid adsorption of methylene blue molecules. But after this stage, the surface of the biosorbent became partially filled, and therefore, the number of available active sites on the surface of the adsorbent decreased [45].

Figure 6e shows that *Nerium* fruit has good adsorption capacities at low concentrations of methylene blue. This trend suggests that large active sites on the adsorbents are accessible at this stage. At high dye concentration, the adsorption capacity reached approximately its maximum. This suggests that the interstitial spaces in the solid adsorbent became more saturated. Indeed, the adsorption occurred synchronously on the surface and in the inner section of the adsorbent. Due to prior contact, adsorption proceeded on the surface during the first stage. Then, the active sites on the surface of the adsorbent reacted with the liquid to reach saturation. Consequently, high dye concentrations could easily saturate the surface adsorption sites. At 19 °C, the maximum adsorbed amount of methylene blue was reached at 259 mg/g. Compared with other studied biosorbents published in the literature (Table 1), this level is too high, and the studied biomaterial could be valorized as an excellent biosorbent to treat colored solutions.

The adsorption of methylene blue using *Nerium* fruit is also affected by a change in temperature (Figure 6e). Results obtained indicate a decrease in methylene blue adsorption capacity with an increase in temperature. This trend is due to the escape of the adsorbed methylene blue ions with increasing energy or temperature.

#### 3.2.2. Kinetic Modeling

**Figure 7:**
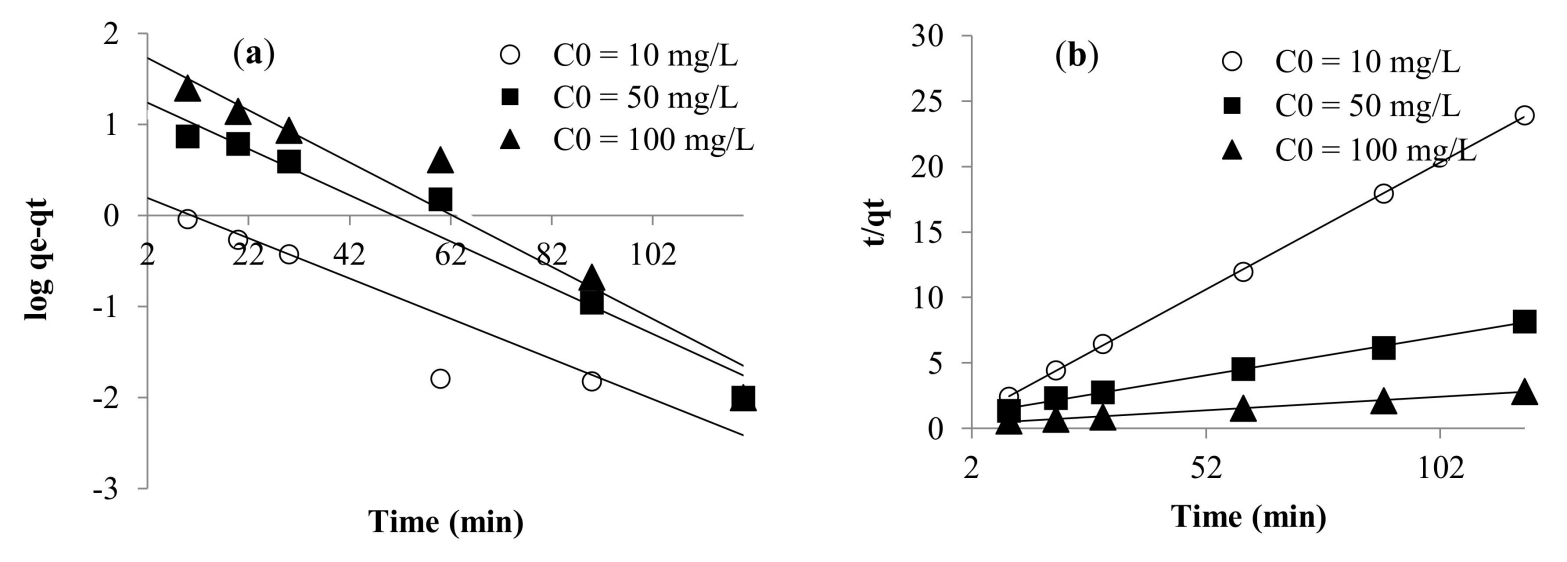

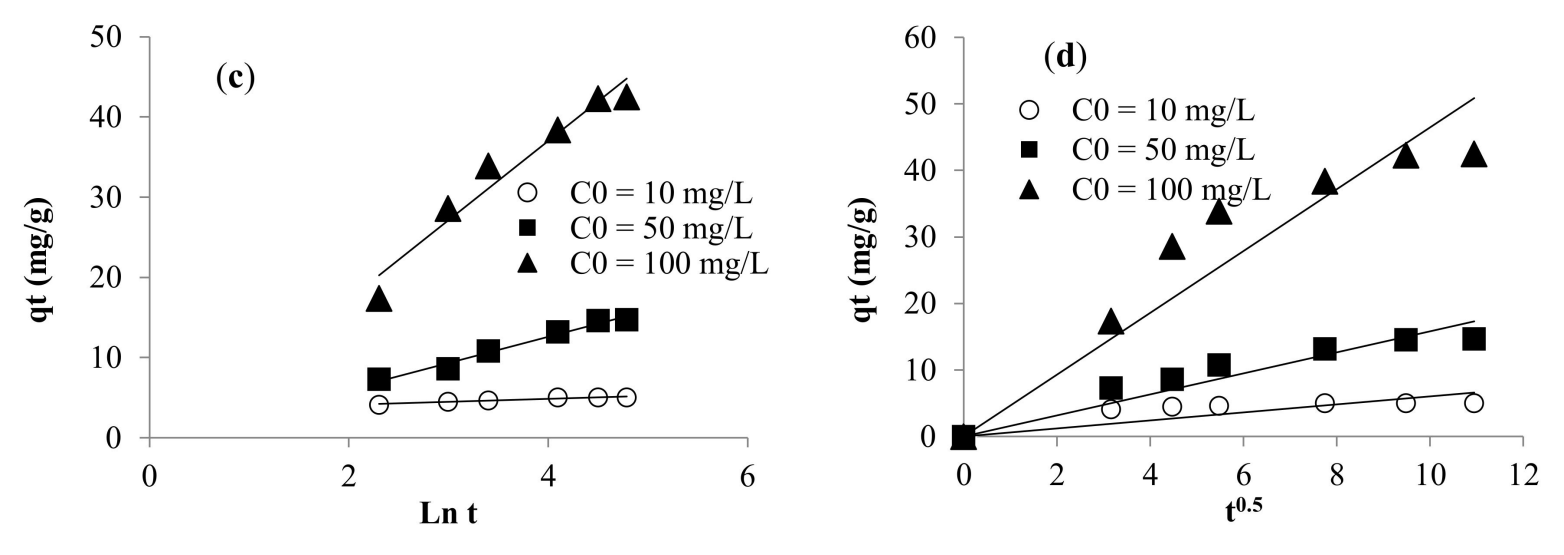
Kinetic adsorption data of methylene blue using *Nerium* fruit linearized through (**a**) pseudo-first-order, (**b**) pseudo-second-order, (**c**) Elovich, and (**d**) intraparticular diffusion equations.

Kinetic data are important to understanding the affinity between methylene blue as adsorbate and *Nerium* fruit as adsorbent at equilibrium. In fact, this study could provide sufficient information on the biosorption mechanism, and it suggests whether it is physical and/or chemical and mass transport. The adsorption of methylene blue using *Nerium* fruit was analyzed using the pseudo-first-order (Figure 7a), pseudo-second-order (Figure 7b), Elovich (Figure 7c), and intraparticular diffusion (Figure 7d) equations. With reference to the obtained curves, the kinetic parameters were computed and are summarized in Table 2. Following the data registered within the pseudo-first-order equation, we observed that the R^2^ values ranged from 0.86 to 0.95. These low values suggest that this equation cannot describe the kinetic data perfectly. Indeed, this equation does not follow well the whole range of time, and it was valid only during the initial stage of adsorption [65]. However, the correlation coefficients obtained within the pseudo-second-order equation were greater than 0.99, which suggests that the adsorption of methylene blue using *Nerium* fruit can be considered a chemisorption process [66]. In addition, the high regression coefficient values (0.94 < R^2^ < 0.98) registered in the case of the Elovich equation suggest a chemisorption with heterogeneous pores on the surface of the adsorbent. The plots for the intraparticle diffusion model are obviously diverged from the origin, suggesting that this equation is not the sole rate-controlling step but that other kinetic processes could occur during the adsorption [67].

#### 3.2.3. Isotherm Study and Thermodynamic Parameter Calculation

**Figure 8:**
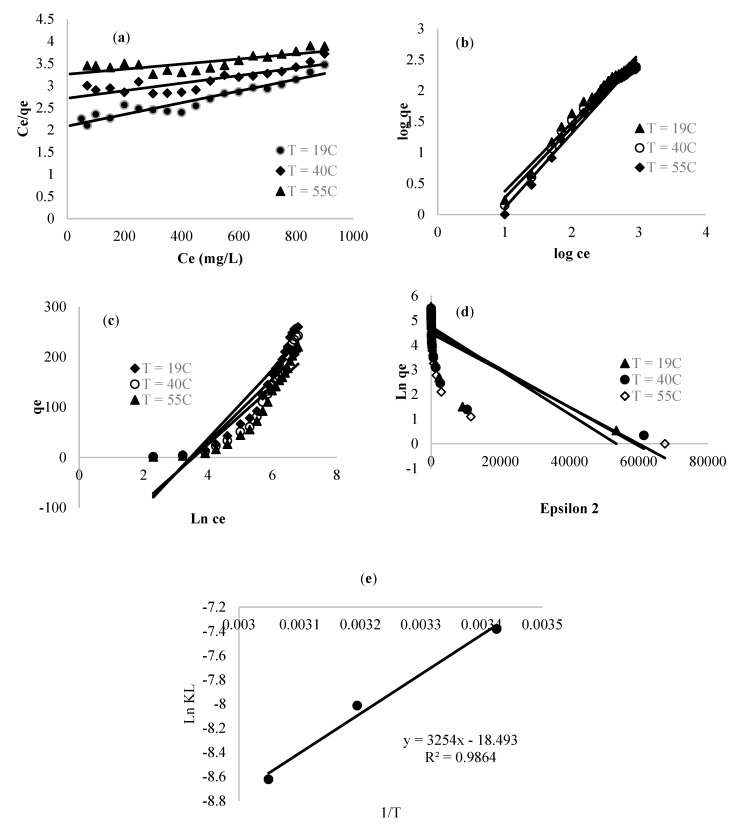
Linearization of the adsorption data of methylene blue using *Nerium* fruit through (**a**) Langmuir, (**b**) Freundlich, (**c**) Temkin, (**d**) Dubinin isotherms, and (**e**) plots of Ln Kl versus the inverse of temperature.

The relationship between *Nerium oleander* fruit and methylene blue was analyzed using the isotherms of Langmuir, Freundlich, Temkin, and Dubinin–Radushkevich (Figure 8a–d). The fitting parameters are summarized in Table 2. As observed, the equation of Freundlich fitted better the adsorption data (0.98 ≤ R^2^) compared with the other studied equations. The consistency of the Freundlich equation with the data revealed that the adsorption may occur in the heterogeneous adsorption sites. The adsorption energy constant (B) determined from the Temkin equation decreased from 68.4 to 57.2 when the temperature rose from 19 to 55 °C. This decrease of the adsorbed quantity of methylene blue with an increase in temperature agrees with the data described above. The values of the heat of sorption were high (bt = 35.48.5–47.69 J/mol), suggesting a chemical adsorption process [68]. The mean free energy values, calculated from the Dubinin equation, ranged from 74.53 to 84.52 KJ mol^−1^. These values are greater than 40 KJ mol^−1^, suggesting a chemisorption mechanism [69]. 

The negative value of the enthalpy (ΔH° = −27.05 kJ/mol) proves that the interaction between *Nerium* fruit and methylene blue is exothermic. This result agrees with the decrease of the adsorbed capacity with temperature. The negative value of the entropy change (ΔS° = −153.75 J/mol) suggests a decrease of the disorder and randomness at the solid–solution interface of methylene blue with *Nerium oleander* clusters. The positive values of the free energy (ΔG° = 17.84–23.37 kJ/mol) indicate that the biosorption mechanism is nonspontaneous.

## 4. Conclusions

The results presented herein focused on the characterization and application of *Nerium oleander* fruit as a biomaterial for the adsorption of methylene blue from aqueous solution. FTIR spectroscopy indicated the presence of the main absorption peak characteristics of cellulose and noncellulose compositions. XRD patterns showed the characteristics of cellulose **I** composition. SEM features showed a rough and heterogeneous *Nerium* surface. TGA analysis indicated that *Nerium* fruit is composed of cellulose and noncellulosic matters. The adsorption capacity reached 259 mg/g at room temperature, which constitutes a high level of capacity compared with other published biosorbents. The values of the mean free energy calculated by the Dubinin isotherm were 74.53 to 84.52 KJ mol^−1^, suggesting a chemisorption mechanism. It was found that adsorption capacity depends on several experimental parameters, including pH, adsorbent dosage, temperature, etc. The thermodynamic study indicates that the adsorption process was chemical, exothermic, and nonspontaneous. Future work will concern the design of biocomposites based on this material for versatile applications. 

## Figures and Tables

**Table 1:** A comparison of the biosorption capacities of methylene blue using some biomaterials published in the literature.

Adsorbent	Target	Maximum Adsorption Capacity (mg g^−1^)	Reference
*Nerium* fruits	Methylene blue	259	Current work
Pineapple stem	Methylene blue	119	[46]
Lotus leaf	Methylene blue	221.7	[47]
Palm tree waste	Methylene blue	39.47	[48]
*Pinus durangensis*	Methylene blue	85	[49]
*Salix babylonica* leaves	Methylene blue	60.97	[50]
Kenaf core fibers	Methylene blue	131.6	[51]
Almond shell	Methylene blue	84.9	[4]
Prickly (peel) bark of cactus fruit	Methylene blue	222	[52]
Algae *Sargassum muticum*	Methylene blue	279.2	[53]
Broad been peals	Methylene blue	192.7	[54]
Algae *Gelidium*	Methylene blue	171	[55]
Rejected tea	Methylene blue	147	[56]
Mango seed kernel	Methylene blue	142.9	[57]
Empty fruit bunch	Methylene blue	50.76	[58]
Rice husk	Methylene blue	40.6	[59]
Orange peel	Methylene blue	18.6	[60]
Jute processing waste	Methylene blue	22.47	[61]
Garlic peel	Methylene blue	82.64	[62]
Date pits	Methylene blue	80.31	[63]
Walnut shell powder	Methylene blue	178.9	[64]
Dead *Typha angustifolia* (L.) leaves	Methylene blue	106.75	[24]
Peach shells	Methylene blue	183.6	[3]

**Table 2:** Summarized kinetic constants, Langmuir, Freundlich, Temkin, and Dubinin parameters for the adsorption of methylene blue using powdered fruit derived from *Nerium oleander*.

Kinetic equations	Constants	Dye concentration (mg/L)			Isotherms	Parameters	Temperature (°C)		
Pseudo-first-order		10	50	100			19	40	55
	K_1_ (min^−1^)	0.022	0.025	0.028		q_m_ (mg g^−1^)	769.23	1111.11	1666.67
	q (mg g^−1^)	1.716	19.40	61.23	Langmuir	K_L_ (L g^−1^)	0.000623	0.000331	0.00018
	R^2^	0.86	0.96	0.95		R^2^	0.91	0.74	0.61
Pseudo-second-order	K_2_	0.0735	0.0037	0.0014	Thermodynamic parameters	ΔH° (KJ mol^−1^)	−27.05		
	q (mg g^−1^)	5.154	16.806	48.309		ΔS° (J mol^−1^)	−153.75		
	h	1.954	1.052	3.245		ΔG° (KJ mol^−1^)	17.84	21.07	23.37
	R^2^	0.999	0.997	0.997	Freundlich	K_F_ (L g^−1^)	0.185	0.135	0.080
Elovich	(mg g^−1^ min^−1^)	2656.25	2.807	7.648		n	0.902	0.878	0.831
	*β* (mg g^−1^ min^−1^)	2.65	0.305	0.1011		R^2^	0.98	0.98	0.98
	R^2^	0.938	0.978	0.948	Temkin	B (J/mol)	68.407	62.665	57.173
Intraparticular diffusion	K (mg g^−1^ min^1/2^)	0.606	1.583	4.645		A (L g^−1^)	0.0314	0.0296	0.0287
						bt (J/mol)	35.48	41.53	47.69
	R^2^	0.339	0.876	0.848		R^2^	0.87	0.85	0.84
					Dubinin	q (mg g^−1^)	115.85	100.35	87.11
						E (KJ/mol)	74.536	79.057	84.52
						K_DR_ (mol^2^/kJ^2^)	9 × 10^−5^	8 × 10^−5^	7 × 10^−5^
						R^2^	0.55	0.54	0.53
